# Analysis and Implementation of Human Mobility Behavior Using Similarity Analysis Based on Co-Occurrence Matrix

**DOI:** 10.3390/s22249898

**Published:** 2022-12-15

**Authors:** Ambreen Memon, Jeff Kilby, Jose Breñosa, Julio César Martínez Espinosa, Imran Ashraf

**Affiliations:** 1 Information Technology, Western Institute of Technology Taranaki, New Plymouth 4310, New Zealand; 2 School of Engineering Computer and Mathematical Science, Auckland University of Technology, Auckland 1010, New Zealand; 3 Higher Polytechnic School, Universidad Europea del Atlántico, Isabel Torres 21, 39011 Santander, Spain; 4 Department of Project Management, Universidad Internacional Iberoamericana, Arecibo, PR 00613, USA; 5 Universidade Internacional do Cuanza, Cuito EN250, Bié, Angola; 6 Department of Project Management, Universidad Internacional Iberoamericana, Campeche 24560, Mexico; 7 Fundación Universitaria Internacional de Colombia, Bogotá 111311, Colombia; 8 Department of Information and Communication Engineering, Yeungnam University, Gyeongsan 38541, Republic of Korea

**Keywords:** similarity analysis, mobility, co-occurrences matrix, device-to-device communications

## Abstract

The fast expansion of ICT (information and communications technology) has provided rich sources of data for the analysis, modeling, and interpretation of human mobility patterns. Many researchers have already introduced behavior-aware protocols for a better understanding of architecture and realistic modeling of behavioral characteristics, similarities, and aggregation of mobile users. We are introducing the similarity analytical framework for the mobile encountering analysis to allow for more direct integration between the physical world and cyber-based systems. In this research, we propose a method for finding the similarity behavior of users’ mobility patterns based on location and time. This research was conducted to develop a technique for producing co-occurrence matrices of users based on their similar behaviors to determine their encounters. Our approach, named SAA (similarity analysis approach), makes use of the device info i.e., IP (internet protocol) and MAC (media access control) address, providing an in-depth analysis of similarity behaviors on a daily basis. We analyzed the similarity distributions of users on different days of the week for different locations based on their real movements. The results show similar characteristics of users with common mobility behaviors based on location and time to showcase the efficacy. The results show that the proposed SAA approach is 33% more accurate in terms of recognizing the user’s similarity as compared to the existing similarity approach.

## 1. Introduction

The IoT (internet of things) is a network where embedded sensors are linked to the web, so that data can be gathered and shared. It allows machines to communicate, cooperate, and benefit from the experiences of others as much as humans do. Since the IoT network generates and analyzes vast amounts of data, it is a major driver of big data analytical projects. It is especially capable of transmitting vast volumes of data in real time. It is possible to improve operations at all locations via various IoT devices; thus, it is an eye-catching research topic for researchers [1]. Researchers have used numerous data tools to research human actions, including bank notes, emails, and GPS (global positioning system) systems [2,3]. Even though data collected from such sources aid in empirically informed human mobility research, such tools also present limitations in size, data resolution, and adaptation [4,5].

Mobile phones have arisen as more effective tools to address the limitations of the tools for behavior analyses [6]. The use of smartphones is growing; they are mainly used for contact and sharing knowledge [7]. An individual typically carries a mobile phone throughout the day. For this reason, the data on mobile phones have attracted the attention of researchers interested in studying the patterns of human mobility [8,9,10]. Handheld agent assemblies with identical characteristics of mobile communities have established trends in wireless networks. This comes as no shock as these features often have significant effects on device behaviors. Researchers have been researching ways to derive and test certain features. One significant point is that periodic re-emergences occur at some sites, which shows the relations between related instances. Therefore, individuals with common concepts of conduct connect; this provides a significant dimension in which pairing of the device position may be used to recognize resemblance trends of smartphone apps. So, to quantify similarity characteristics among cell phones for this study, we used their interests in spatial, temporal, and preferred word attachments to the sites and the travel frequencies and lengths of those areas [11,12].

The existing approaches allow for the use of values dependent on the venue. Such ideals have common perspectives of sites at somewhat remote positions, although those sites can share the same network in most situations. Even some existing strategies have used the associated matrix and multiple access points for each building, so the building’s doors and rooms are distinct locations, and people at such locations are assumed to have different mobility behaviors rather than identical mobility behaviors [5]. In this paper, we used the co-occurrence matrix to capture human mobility preferences. In the co-occurrence matrix, we used the location and time. We consider the location as a single access point for each building. We used device info, i.e., IP and MAC addresses, to identify the user’s time and location. When different people are in the same building and are slightly apart, i.e., at different doors of a building, then the proposed approach regards these devices as having the same mobility behavior; instead of using different networks for data transmission, the same network is used to show similar mobility behavior. Our suggested approach involves examining correlation patterns in detail on a regular basis.

The remainder of the paper is structured as follows. Section 2 presents the literature review. In Section 3, we provide the methodology, system model for similarity analysis, the proposed co-occurrence matrix to capture the human mobility preferences, as well as an introduction of the similarity analytical framework for the mobile encountering analysis. We conducted a case study. In Section 4, the results are discussed. We highlight the comparison results of the ‘SAA’ approach with the existing work. In the final section, the conclusions and future work are presented.

## 2. Related Work

Human behavior involves common perspectives of sites in somewhat remote positions; those sites can share the same network in most situations. Even some existing strategies have used the associate matrix and use multiple access points for each building, the building’s doors and rooms are taken as distinct locations even if they are at the same location [5]. To study human behavior, researchers have employed various data resources, including bank notes, tweets, and GPS devices [13]. These data can be useful for traffic control [1], city planning [14], mobile marketing [15], and much more. The data obtained from these sources can also help in empirically driven studies on human mobility. However, these resources show limitations in scale, data resolution, and adaption. To overcome the constraints in behavioral analysis resources, mobile phones have emerged as more efficient resources [16].

The use of smartphones is increasing; smartphones have become essential parts of our lives. The mobile phone is mostly used for communication and information exchange. People typically carry mobile phones around. The data used by a mobile phone is another way of representing the mobility preferences of a mobile user. Hence, it is a useful tool for identifying human mobility patterns. For this reason, mobile phone data are attracting researchers who are interested in studying human mobility patterns. As with mobile data, a large number of users can be tracked in the long term at a low cost. Among mobile users, some show similarities in mobility behaviors while others are dissimilar to one group but similar to another. This is what we call similarity in mobility behavior. Before discussing the similarities in mobility behaviors, first, we shall focus on the existing models for mobility modeling. Mobility modeling provides a medium for simulating mobility in wireless ad hoc networks [2,17], urban planning, and disaster response systems. Mobility models use mobility traces to understand the nature of mobility. In mobility modeling, we usually collect the real-time data of mobile users carrying a mobile phone with them during traveling, shopping, in the office, or university, etc. The mobility traces are the results of human mobility. This mobility is a result of repeated movements of a user to a specific place, time-based human activities, mobility, and mobility based on social relationships [2].

The mobility of mobile users may be at a large scale, e.g., traveling from country to country, or by airplanes and trains. Mobility at a smaller scale can involve, for example, traveling within a city or a university, or from home to an office. After the collection of traces, the data are altered and the daily life environment can be modeled. Moreover, mobility patterns can be used to model people’s decisions based on their behaviors. Through mobility modeling, various operations of a network, i.e., resource management, routing, handover, and even a better independent deployment of connectivity models, can be simulated. The simulation is possible due to the possibility of understanding human movement patterns and regularities [14]. Modeling human mobility, at first, understanding the mobility preferences of people is required. Mobility traces gathered through GPS devices, social media accounts, and mobile phone networks can be used to analyze human movements deeply at large scales. Moreover, there lies a high degree of predictability in human mobility as most people tend to spend most of their time at specific locations. The time to visit a particular location is also mostly fixed [17].

Although preliminary research suggests a connection between mobility, activity data, and clinical phenomenology, no further conclusions can be drawn given the limitations of the methods that are currently used. For instance, a 2015 study found a correlation between the severity of depressive symptoms and GPS-derived metrics, such as normalized entropy and location variance [18]. However, the same group conducted a subsequent study by controlling multiple comparisons and found less conclusive results [19]. The study highlights the complex relationship between depression and geolocation data; schizophrenia likewise presents disconnected results with portability measurements sometimes related to fading psychosis [20,21], varying with other studies [22]. We propose that difficulties in utilizing the data rather than any analysis are to blame for the lack of consensus.

Decomposing the smartphone sensor data into time series metrics and transforming them into clinical features are two major problems. First, most studies do not replicate because they make use of custom rules for extracting various features, such as sleep duration. Indeed, even Apple frequently changes its element calculations, making replications utilizing Apple wellness information more complex [23]. Even though some features must be built from raw accelerometer data, such as movement bouts, converting that data into other derived metrics, such as running, sitting, and standing frequently involve complex tasks. To make use of the time series data that comes with smartphone data capture, the challenge of breaking down the data series nature into temporally aligned parts is crucial. However, these kinds of data are rarely used in today’s research, with many papers utilizing summary metrics as data endpoints [24]. Even though these data are valuable, clinically relevant information is lost or ignored.

## 3. Similarity Analysis Approach

In this section, we calculate the similarity analysis with the co-occurrence matrix using the capturing spatial–temporal values and user pair analysis. The calculating similarity consists of the following steps:

- Matrix information based on an IP (internet protocol) address;
- Capturing spatial–temporal values for the co-occurrence matrix;
- Similarity analysis with the co-occurrence matrix.

### 3.1. Information Based on IP Address

A user’s mobility in different places causes it to connect to different networks. This connection with the networks assigns mobile devices to separate IP addresses. An IP address is a 32-bit long address assigned to each device when it connects to a network. An IP address can also provide geographical information about a device [25]. An IP address has two parts: a network ID and a host ID. The network ID shows the information on the type of the specific network to which the device is connected. The host ID provides information about the particular host in the network. In comparison, the MAC (media access control) address is a 48-bit unique identifier of each device.

**Figure 1:**
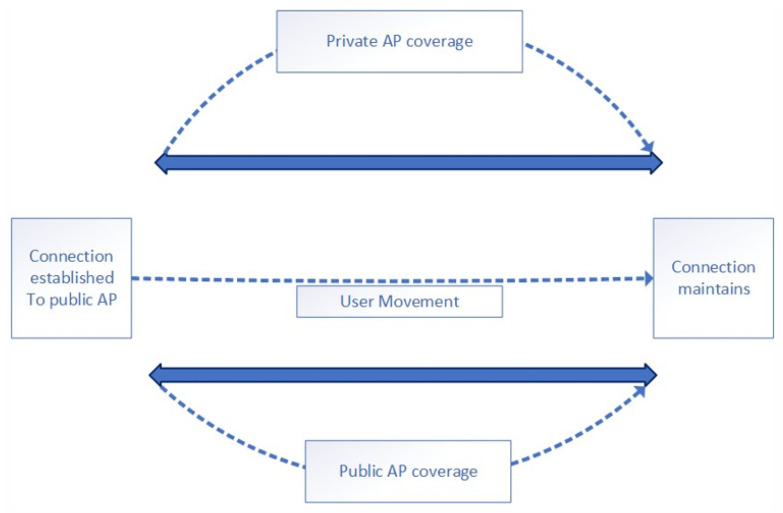
Mobile user connectivity behavior with access points.

In recent research, Kim et al. [26] demonstrated that if a user is connected to an AP (access point), the user keeps the connectivity unless the AP goes out of range and only then connects to the new best available AP. In Figure 1, a user is connected to a public AP, and even when the user arrives home, a better connection is available. The user stays connected to the less reliable/slow public network unless the user himself shifts to the better network or the public AP goes out of range. In a similar analogy, a set of two users, despite having a small Euclidean distance between them, might be connected to a different set of APs, and have different connectivity routes. This gives rise to the motivation that IP-based similarity has been used rather than a Euclidean distance-based user’s mobility similarity analysis.

By keeping the scenario already mentioned above, in the presented research, the AP connectivity is kept as a sign of presence at a location, i.e., if two users are connected to the same AP are considered to be at the exact location. This increases the chance of the two users having a direct connection in the MANET (mobile ad hoc network) scenario. This connectivity consideration helps us better correlate the user’s mobility patterns and shows improved similarity.

The proposed method models the space–time associations of the mobility patterns and is, thus, better able to determine the similarities among the mobility patterns of users. a user’s presence probability at a location is used to find the co-occurrence with others as part of the model. In the proposed model, we used cosine similarity as a measure to estimate the correlation among users. If the mobility patterns are correlated, i.e., mobility directions are the same, then the cosine similarity enables the model to compute similarity patterns among the users. Here, it is important to mention that the IP information is central to determining the geo location of a user. As previously mentioned, the IP address not only shows the device ID but network information as well. In the model, IP addresses are used as a whole, which results in the construction of a more informed model and, hence, better similarity patterns are obtained. In Equations (1) and (2), the joint probability of the user’s presence based on the IP address is made part of the model. The model is explained in the next section and the effectiveness of the model is verified from the results presented later.

### 3.2. Capturing Spatial–Temporal Values for Co-Occurrence Matrix

The representation of IP address (location) and time values are done in form of a co-occurrence matrix. In this co-occurrence matrix, each row vector describes the normalized percentage of time the user spends at each location on a measured time, e.g., from 8 a.m. to 8 p.m. each day. The column reflects the most significant locations of the user. Depending on the cases, we can define the granularity of the time duration as an hour, a day, or a week. It is similar to the location, as a specific building in which the matrix provides flexibility to represent the problems.

### 3.3. Similarity Analysis with the Co-Occurrence Matrix

This section explains mathematical notations used to describe the model. *P* is represented as an occurrence matrix with the order as m×n. The *n* denotes location (each building has its access point), and *m* represents a particular day. Let $p_{ki}$ define the specific time in the *k*th row at the *i*th is the location of *P*, where *M* denotes the occurrence matrix of user 1…n, *M* is a matrix in the order of m×n. Let $M_{ij}$ represent the time in rows, and *J*th the location of *M*. For *i* is not equal to *j* when $M_{ij}$ equals the number of co-occurrences of the time and location, *I* and *j*.

(1) $$M_{ij}=\sum_{k=1}^{m}p_{ki}p_{kj}$$

Suppose $T_{j}$ denotes the total object occurrence or object co-occurrence at *j* (location).

(2) $$T_{i}=\sum_{k=1}^{m}p_{ki}$$

Suppose the total object occurrence or object co-occurrence at *j* (location) is denoted by $T_{j}$.

(3) $$T_{j}=\sum_{k=1}^{m}p_{kj}$$

#### Direct Similarity Measure

The $F_{c}$ shows the ratio between the observed time of users *i* and *j*, separately. The cosine angle is used between the *i*th and *j*th columns of the co-occurrence matrix of *P* for measuring.

(4) $$F_{c}=\left(M_{ij},T_{ij}\right)=\frac{M_{ij}}{\sqrt{\left(T_{i},T_{j}\right)}}$$

In the above-given model, the concept of cosine similarity is used to show the similarity of patterns in the mobility traces of the users. The user’s movement pattern is considered a vector and the angle between the vectors is inversely proportional to the similarity between the vectors, i.e., the mobility traces of the users. The inherent idea of cosine similarity is that the vectors point in the same direction and have a higher similarity irrespective of their Euclidean distance. Here, if two users follow a similar movement path, then they would have a higher similarity. The similarity is modeled as a probability in this context.

### 3.4. Benchmark for Similarity Analysis

The existing work for the similarity analysis uses longitudinal wireless activity sessions to build a mobile user’s spatiotemporal profile [5]. The representation of spatiotemporal preferences in the form of an association matrix can be changed to use each column for a building (where the collection of access points represents a building) and the time granularity can be changed to represent hourly, weekly, or monthly behaviors. In the existing work, each row represents the day in the traces and the column represents the access point. After that, it calculates the SVD (singular value decomposition) of each associate matrix. The SVD of a given matrix *A* can be represented as a product of three matrices: an orthogonal matrix *U*, a diagonal matrix *S*, and the transpose of an orthogonal matrix *V*. It is written as

(5) $$A=U.S.V^{t}$$

After that, a mobile encounter index metric is represented $M_{ei}$, which indicates the similarity ranking (i.e., 0 represents totally dissimilar and 1 indicates exactly the same) between two objectives’ mobility preferences by quantitatively measuring the similarity between the eigenvectors of their mobility matrices. For the pair of objectives, with respective eigenvectors as $A=a_{1},a_{2},a_{3},\cdots ,a_{ar}$, and $B=b_{1},b_{2},b_{3},\cdots ,b_{rb}$, the mobility similarity can be calculated by the weighted sum of the pairwise inner product of their eigenvectors as

(6) $$M_{ei}\left(A,B\right)=S_{AB}=\sum_{i=1}^{RankA}\sum_{i=j}^{RankB}W_{ai}W_{bj}\left|A_{i}B_{j}\right|$$

where $M_{ei}\left(A,B\right)$ is a quantitative measure index that shows the closeness of two mobile peers in their mobility preferences in the time–space dimension. $S_{A,B}$ is an index that shows the closeness of users in terms of the values in their co-occurrence matrix. Here, *A* and *B* are the respective eigenvectors of the users. $W_{ai}$ and $W_{bj}$ are the numbers of co-occurrences of users *A* and *B* at specific locations. Co-occurrence is measured in terms of time, location, and device information.

The similarity analysis shows that the value of the similarity is within the range of $0\leq S_{A,B}\leq 1$. The lower similarity rank indicates that peers have very different time–space mobility preferences. In other words, they have fewer opportunities to meet each other somewhere. Otherwise, for the higher similarity-ranked cases, the user pairs have similar mobility preferences, or they have high probabilities to meet each other in some locations where they have the same interests to visit. In addition, we can also quantify how the mobility preferences similarity between the same pair of moving objectives varies with time. Firstly, we can calculate the similarity between two mobile objectives, e.g., *A* and *B*, at two points in time, $M_{ci}{\left(A,B\right)}_{T1}$ and $M_{ci}{\left(A,B\right)}_{T2}$, where *T* represents a specific time instance. We perform this calculation on all user pairs and investigate the relationship between the users’ location and time. We calculated the MCC (mobility correlation coefficient) of the similarity matrices obtained after a *T* interval as

(7) $$M_{cc}=\frac{\sum_{A,b}\left(X-\bar{X}\right)\left(Y-\bar{Y}\right)}{NS_{x}S_{y}}$$

where $X=S_{A,B}\left(T1\right)$, $Y=S_{A,B}\left(T2\right)$, and the notations $\bar{X}$ and $S_{X}$ denote the average and standard deviation of *X*, respectively. *N* is the total number of mobile user pairs. The mobility correlation coefficient indicates how stable the interactions between the peer pairs are. It has been reported that the similarity metrics between objective pairs correlate reasonably well if the considered periods are not far apart.

### 3.5. The Case Study for the SAA Approach

This section contains a case study for understanding the SAA approach and its work in detail. We conducted a case study where the data transmission distance is a key but changeable factor contributing to overall energy consumption. It is straightforward that the shorter the transmission distance is, the more the total energy consumption will be reduced. For example, we have two users, Alice and Bob, working at the same university but on different campuses. Alice is a professor at the university, and Bob is studying there; they normally have to meet to discuss the research work. Alice intends to send a file to Bob, which will be required by Bob after seven days. At the moment, Bob is working on another task. In this case, how can Alice send the file to Bob? There are several options available:

Option A: Alice can send the data through the internet in a traditional way.
Option B: A direct file-transfer service can be used; for example, Alice and Bob can exchange the data through point-to-point transfer through routers.

Options A and option B are the most popular ways; overall energy consumption depends upon the distance and the size of the data. If we know Alice and Bob will meet, we can consider option C. We have fewer requirements of time, data are not more urgent, and the delay-tolerant indicator is decided to reach the data between that duration. In option C, human mobility traces are matched according to behavior. Alice and Bob have similar mobility preferences as they are at slightly distant locations (on any floor/room of the building). They are also connected to the same network.

## 4. Numerical Studies

### 4.1. Dataset

This section provides the details of the traces that have been used for the experiments. The traces are real measurements taken from the USC (University of Southern California) [27]. The data contain information about Wi-Fi associations and user profiles. The data involve different buildings on the campus, as well as access points, and are collected on different days, including weekdays and weekends. For this study, we chose campus environments since they are comprehensive with a high number of active users, and are associated with sufficient location samples. The data consist of Wi-Fi usage and location information.

### 4.2. Simulation Experiment

We abstracted a subset of peers that are independent of each other (as much as possible) from the large population data. Then we conducted a relevant statistics analysis on the selected co-occurrence matrix data of mobile peers. This matrix consists of information about the AP, temporal values, IP address, and MAC address of the mobile devices. The data set was divided based on each day, i.e., for seven days a week. For each day, we used iterator tools for bringing the data in a sorted form, i.e., all combinations were extracted. The locations visited by both users, *x*, *y*, were sorted and their co-occurrences were recorded. After recording multiple co-occurrences, we removed the redundant information and only unique location IDs were extracted, such as iterator tools (functions creating iterations for efficient looping) and redundant information (using group by command).

### 4.3. Simulation Results

**Figure 2:**
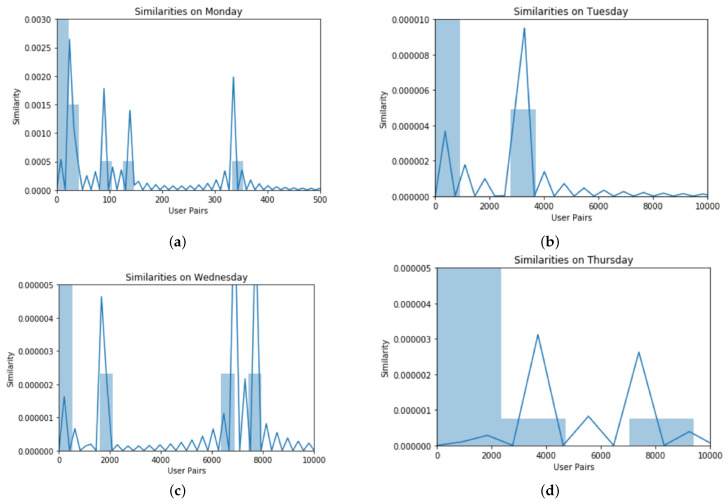
Similarity analysis during different days, (**a**) On Monday, (**b**) On Tuesday, (**c**) On Wednesday, and (**d**) On Thursday. Similarity shows that these users are found on the same location during the same time frame.

**Figure 3:**
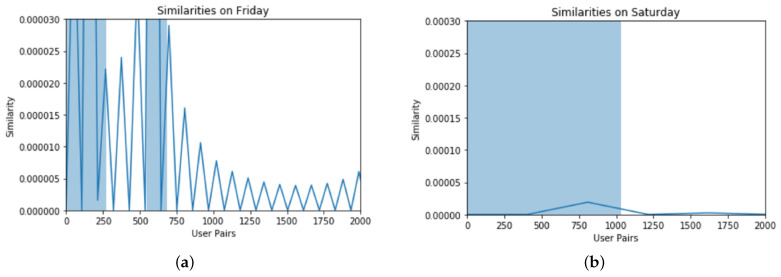
Similarity analysis during different days, (**a**) On Friday, and (**b**) On Saturday. Similarity shows that these users are found on the same location during the same time frame.

Based on the analysis of similarity profiles, we found the following results. At first, different user pairs are shown that are at specific locations of the same time span. For calculating mobility similarities among different user pairs at different time intervals, data of the users are selected from the USC traces available in the dataset. Figure 1, Figure 2 and Figure 3 show different pairs of users who are found at the same location at the same time interval. The following histograms show the similarities of different user pairs on different days of the week.

The users are from the same data set that we used for experiments. In these histograms, the number of user pairs is taken as a function of similarity showing the behavioral similarity of different days of mobile users. The result was a co-occurrence matrix having no null value and combinations of visited locations for both users. Then we deployed the SVD technique on the co-occurrence matrix for a normalized output. Each similarity value was calculated for different days and plotted respectively. The experiment was performed to show that the SAA approach is a deep analysis (on a daily basis) of similar behavior. Figure 1, Figure 2 and Figure 3 show the deep analysis of different pairs of users that are calculated at the same location at the same time interval. The following histograms show the similarities of different user pairs on different days of the week. The users are from the same data set that we used for experiments. In these histograms, the number of user pairs is taken as a function of similarity showing the behavioral similarity of different days of mobile users.

The data set was divided based on each day i.e., for seven days a week. For each day, we used iterator tools to bring the data in sorted form, i.e., all combinations were extracted. The locations visited by both users *x* and *y* were sorted and their co-occurrences were recorded. After recording multiple co-occurrences, we removed the redundant information and only unique location IDs were extracted. We used iterator tools that were function-creating iterations for efficient looping and redundant information using groups by command.

**Figure 4:**
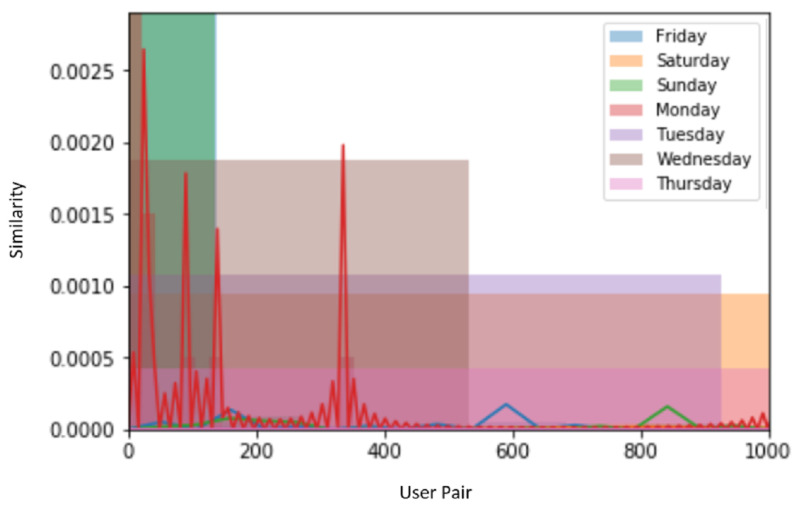
Similarity analysis of all 7 days of a week.

For weekends, i.e., Saturday and Sunday, the lowest similarity reached zero and the highest similarities were 0.000020 and 0.00008 as the number of active users was quite less due to the absence of academic activity. In Figure 4, we compared the similarity characteristics of those users who have common mobility behaviors based on location and time. The comparative analysis of 7 days shows a very high similarity on Monday.

The comparison results show a similar pattern with the increased number of similarity–user pairs in SAA as shown in Figure 4. The *x*-axis shows similarity and the *y*-axis shows user pairs.

**Figure 5:**
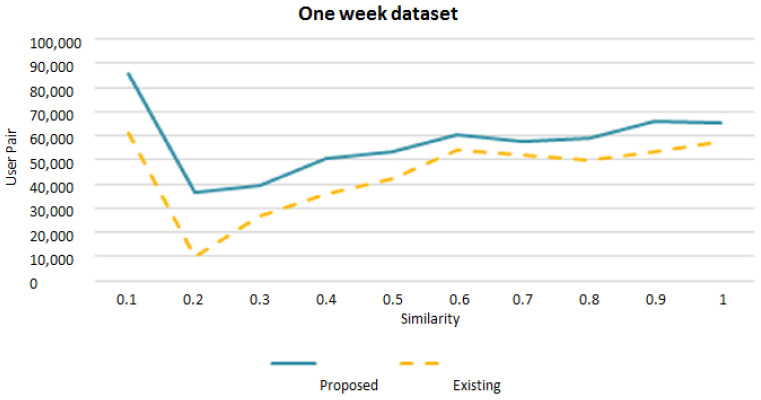
SAA approach similarity comparison with existing work.

The graph given in Figure 5 shows the comparison of our purposed mode SAA with the existing work. The existing approach uses longitudinal wireless activity sessions and assigns more than one access point to a building and considered a different location. Whereas our model is based on location and time with one access point to each building, and the building is considered as one location. To capture more mobility patterns, we used the co-occurrence matrix. The comparison results also show that the proposed model SAA is 33% more efficient than the existing approach. The comparison results are based on (a one-week) dataset.

## 5. Conclusions

A mobile phone is the most suitable and cost-efficient way to analyze mobility behavior. This study provides a similarity analysis approach for finding behavior similarities in user mobility (using mobile phone data). We used the spatial–temporal parameters of a mobile user and the device network information of the mobile node in this regard. We provided the spatial preferences of a user and temporal values at which particular locations were visited. For experiments, we used the mobility traces of users from different campuses, i.e., (campus1, …, campus6). After extracting the spatial–temporal associations of traces, we stored them in the co-occurrence matrix and normalized the time associations, and applied the SVD technique to extract the preferred locations and times of a mobile user. During the experiments, we performed a similarity analysis of the processed data and calculated the similarities of different users on different days and campuses. The results show the similarity characteristics of users with common mobility behaviors based on location and time to showcase the efficacy. The results show that the purposed approach is a 33% more accurate behavioral model as compared to the existing similarity approaches. In the future, we plan to further investigate similarity modeling and prediction for similarity analysis. We also intend to add more dimensions for a comparison analysis, such as response time, energy consumption, etc.

## Data Availability

The dataset and code is available from the corresponding author on reasonable request.
